# Safe from sunburn: The divergent diel pattern of a *Hydrophis* sea snake

**DOI:** 10.1002/ece3.8436

**Published:** 2022-01-24

**Authors:** Brooke Bessesen, Manuela González‐Suárez

**Affiliations:** ^1^ Ecology and Evolutionary Biology University of Reading Reading UK

**Keywords:** activity levels, circadian rhythms, Costa Rica, endemic, ethology, marine snakes

## Abstract

Diel activity patterns are an important aspect of wildlife ecology and evolution and provide valuable information for conservation and monitoring, yet for many species, activity patterns remain unstudied and may be presumed to mirror related taxa. Here, we describe the distinct diel patterns of an endemic population of venomous sea snakes *Hydrophis platurus xanthos* inhabiting a narrow range (circa 320 km^2^) in Golfo Dulce, Costa Rica. To investigate, we conducted a systematic visual survey over five 24‐h cycles and evaluated 339 h of previously obtained sighting data from different studies spanning a decade. While sporadic diurnal surfacing does occur, mostly for respiration, our observations revealed marked crepuscular peaks with regular surfacing through the night. We also report on observed surface behaviors that were also found to vary in frequency at different phases of the photoperiodic cycle. In particular, we show feeding as more common at night. *Hydrophis platurus xanthos* has developed a circadian rhythm that differs noticeably from its taxonomic parent (*H. p. platurus* is reported as diurnal across its Indo‐Pacific range), and no congeners have been categorized as crepuscular. Our work thus contributes to the ecological knowledge of this evolutionarily distinct marine elapid and offers insights into the potential role of environmental conditions in shaping animal activity.

## Graphical abstract

We investigated the activity and behavior patterns of the xanthic sea snake, *Hydrophis platurus xanthos*, which is endemic to the warm fiord‐like embayment of Golfo Dulce, Costa Rica. Data obtained from a systematic visual survey over five 24‐h cycles along with sighting data from several previous studies showed *H. p. xanthos* to evince a nocturnal diel pattern with marked crepuscular peaks. As this circadian rhythm differs noticeably from its taxonomic parent (*H. p. platurus* is reported as diurnal across its Indo‐Pacific range) and no congeners have been categorized as crepuscular, our work offers insights into the potential role of environmental conditions in shaping animal activity.

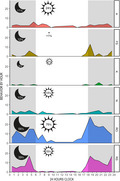

## 1 INTRODUCTION

Animal activity patterns are an important aspect of ecology and evolutionary biology, yet they are an understudied facet of behavioral ecology due to the challenges of recording and quantifying the behaviors of free‐ranging populations over time‐specific periods (Bridges & Noss, 2011). Four biological clocks are triggered by changing environmental conditions that predictably repeat at regular intervals: circadian, tidal, lunar, and seasonal. These clocks influence animal activity patterns, the most obvious of which may be seen on the diel scale (Aschoff, 1984; Pittendrigh, 1981). It has been suggested that an internal timer, often referred to as a “circadian clock,” syncs with changes in the animal brain and/or other parts of the central nervous system that effect a variety of physiological functions and behaviors. The most ubiquitous zeitgeber, or cue, for these circadian rhythms is the daily light‐dark cycle, which includes both the principal periodic states (day: light, night: dark) and the transitions between those states (twilight; Aschoff, 1954, 1984). Temperature is another primary driver (Edery, 2000), and thermoregulation through exposure to or protection from solar radiation is a well‐studied aspect of circadian rhythmicity (Angilletta, 2009). Selection pressure favors organisms that conform to the most beneficial periodicities allowed by their environment (Cloudsley‐Thompson, 1960), and wild populations are generally adapted to diurnality (active during the day), nocturnality (active at night), crepuscularity (active during twilight), or cathemerality (active at irregular hours; Vazquez et al., 2019), although such categories do not account for more subtle shifts in activity levels through the 24‐h cycle.

Activity is usually meant to denote movement (Aschoff, 1954), observed as periods of foraging, traveling, or reproductive behaviors juxtaposed to periods of stillness (resting or sleeping). Certain fauna, however, such as air‐breathing marine snakes that must regularly surface to ventilate, may not present such defined periods. The wide‐ranging pelagic sea snake, *Hydrophis platurus platurus*, spends 87% of its time submerged, either descending or ascending in the water column (Rubinoff et al., 1986). Subsurface swimming occurs at a slow rate of 2–4 cm s^−1^ (Graham et al., 1987), and long dive cycles keep the animal in a near‐constant state of movement. Meanwhile, many vital behaviors take place at the ocean surface with minimal physical activity, including feeding (opportunistically capturing small fish from a floating position; Brischoux & Lillywhite, 2011; Klauber, 1935), hydration (drinking from freshwater lenses after rainfall; Lillywhite et al., 2012, 2019), parturition (live birth: true sea snakes are viviparous; Greene, 1997), pulmonary ventilation (every 37 min on average; Rubinoff et al., 1986), and possibly resting in the conventional sense (episodes of relative stillness; personal observation). Therefore, “diel surfacing patterns” rather than “diel activity patterns” may be more appropriate for interpreting the behavioral ecology of this species and for monitoring purposes. Across its entire Indo‐Pacific range, *H. p. platurus* is described as diurnal, spending more time at the ocean surface (often in drift lines: Kropach, 1971; Lillywhite et al., 2010; Tu, 1976) during daylight hours, a deduction based upon field observations (Brischoux & Lillywhite, 2011; Kropach, 1975; Lillywhite et al., 2015; Rubinoff et al., 1986), ocular structure (Lillywhite, 2014), and optical genetics (Simões et al., 2020). Other *Hydrophis* species were categorized by Simões et al. (2020) as diurnal, nocturnal, or cathemeral, but none as crepuscular.

In opposition to the diurnality of *H. p. platurus*, its only known evolutionary descendent and the subject of our research, *Hydrophis platurus xanthos*, has been suggested to exhibit nocturnal surfacing (Bessesen, 2012, 2015; Lillywhite et al., 2015), although no systematic diel studies had been conducted, and those preliminary data were hindered by low or no sampling efforts for certain times. *Hydrophis platurus xanthos* is endemic to the inner‐basin waters of Golfo Dulce, Costa Rica, an area with higher sea surface temperatures (SST) and lower salinity than those found in the neighboring Eastern Tropical Pacific Ocean (Rasmussen et al., 2011; Rincón‐Alejos & Ballestero‐Sakson, 2015; Wellington & Dunbar, 1995). This allopatric population has transitioned from a black‐backed, yellow‐bellied phenotype to monochromatic xanthic (yellow) coloration and smaller body size (Bessesen & Galbreath, 2017), presumably to avoid overheating under solar exposure (Bessesen, 2012; Solórzano, 2011). Though, with numerous avian predators in Golfo Dulce (personal observation), such conspicuous coloration and lost countershading could negate certain survival advantages. Phenotypic adaptations are often associated with behavioral shifts (Lukoschek & Keogh, 2006; Shetty & Shine, 2002), and a change in circadian rhythm may be another consequence of occupying a warmer habitat and any related morphologic changes.

Diel studies of wild animals were historically conducted via direct observations (Belovsky & Slade, 1986) and often bore challenges such as concealing observer presence to avoid disruption of natural behaviors and ensuring sufficient visibility at night. For land‐based studies, the development of tracking collars and then camera traps that could autonomously detect and capture animals in action without the disturbance of human presence (Bridges & Noss, 2011) provided new opportunities for the statistical modeling of animal activity patterns and increased contributions to this growing field of study (Distiller et al., 2020; Lashley et al., 2018; Rowcliffe et al., 2014; Zhang et al., 2017). Camera trap studies with terrestrial snakes suggest adequate performance for faunal detection (Neuharth et al., 2020) but may be less effective for specific ethological investigations (Welbourne et al., 2017). Camera traps in marine environments are even more problematic. Baited remote underwater video stations (BRUVS) attached to the sea floor have been used to assess sea snake presence in the Great Barrier Reef (although diel patterns were not described; Udyawer et al., 2014). However, securing cameras at the ocean surface in waters <200‐m deep, to sample a small, rarely seen marine snake that produces minimal lateral movement across a study area of several hundred square kilometers, impaired by waves, weather, and low‐light conditions is logistically untenable. Other sea snake studies have employed transmitters (Burns & Heatwole, 1998; Shetty & Shine, 2002), including Udyawer et al. (2015) and Udyawer et al. (2017), who used surgically implanted transmitters in two different *Hydrophis* spp. off Australia to study their fine‐scale diel patterns, even applying accelerometry to decipher behavioral activities (Brown et al., 2013). Rubinoff et al. (1986) and Rubinoff et al. (1988) worked specifically with *H. p. platurus*, suturing dive tags to the outer skin of 15 individuals off Panama and following them with a hydrophone to trace their movements. Transmitter studies have substantial scientific value, but small sample size may limit inference about the population (Lindberg & Walker, 2007), unnatural behavior can bias data (Fitch & Shirer, 1971), and the methods may negatively impact captured and tagged individuals (Riley et al., 2017; Rudolph et al., 1998; Weatherhead & Blouin‐Demers, 2004; Wilson & McMahon, 2006). Thus, to systematically examine the diel‐surfacing patterns of *H. p. xanthos*, we opted for real‐time around‐the‐clock visual observations analyzed with circular statistics. During our observations, we also classified various surface behaviors that allowed us to consider other behavioral rhythms. Our results help fill several knowledge gaps, adding to the literature on behavioral ecology in sea snakes and the adaptive evolution of geographically isolated organisms, while demonstrating the potential for dynamic diel patterns to be recorded on a population scale.

## 2 MATERIALS AND METHODS

### 2.1 Study subject

*Hydrophis platurus xanthos* (Bessesen & Galbreath, 2017) is currently listed as a subspecies of the venomous pelagic sea snake *H. p. platurus*. Apparently allopatric and geographically bound to the Costa Rican embayment known as Golfo Dulce, with an estimated range of about 320 km^2^ (Bessesen, 2012), *H. p. xanthos* is an inherently rare and vulnerable taxon (Drever et al., 2012; Rabinowitz, 1981). Xanthic coloration and small body size (average 49 cm long and 47 g in weight) presumably increase fitness in its relatively warm environment (Bessesen & Galbreath, 2017), but in addition to morphologic adaptations, *H. p. xanthos* exhibits distinct behavioral differences (Bessesen, 2012; Lillywhite et al., 2015). Like its conspecifics, *H. p. xanthos* eats small fish that gather at the surface; prey is secured with a sideway strike of the head, envenomated and swallowed whole. However, by and large, feeding appears to occur at night, when the snake frequently assumes a unique sinusoidal ambush posture with its head directed downward (Bessesen & Galbreath, 2017). Despite the ability to swim both forward and backward, this sea snake never travels more than a meter or two across the sea surface except through passive drift (personal observation). Unlike its evolutionary parent, this taxon shows no association with drift lines (Bessesen, 2012; Lillywhite et al., 2015).

### 2.2 Study site

Our diel survey took place in the inner basin of Golfo Dulce, Costa Rica (centered at 8°37′N, 83°19′W). In this northern sector of the 50‐km‐long embayment, waters up to 215‐m deep are held by steep coastal slopes and a 60‐m sill. In addition to the protective bathymetry, the inner basin is sheltered by the geographic shape of Golfo Dulce, which hooks strongly to the left, further limiting the effects of oceanic currents and hydrographic exchange (Svendsen et al., 2006). As a tropical region, the climate is bimodal, with seasonality based on monthly precipitation: dry (<300‐mm rainfall, December–April) and rainy (>500‐mm rainfall, May–November; Morales‐Ramírez et al., 2015). Air temperatures are generally warm, averaging 21–33.5°C (Lobo et al., 2008). Combining dry and rainy season data, SST in Golfo Dulce can average ~30°C (Rincón‐Alejos & Ballestero‐Sakson, 2015), compared with an average of ~28°C in the neighboring Eastern Tropical Pacific (Rasmussen et al., 2011; Wellington & Dunbar, 1995). Conversely, salinity in Golfo Dulce is lower at ≤31.9 ppt (Rincón‐Alejos & Ballestero‐Sakson, 2015) compared with the oceanic standard of 35 ppt, probably due to the influx of freshwater from four large rivers and numerous tributaries (Wolff et al., 1996). Annual day‐length variation in Costa Rica is minimal (~1 h; Rivera & Borchert, 2001), and during the diel survey, the photoperiod only changed by 15 min, with sunrise shifting from 05:39 h to 05:24 h and sunset remaining constant at 17:50 h.

### 2.3 Field protocols

In a camera trap study, the sensor (camera) remains stationary, and the animal makes lateral movements past the lens, but because *H. p. xanthos* does not swim notable distances at the surface, we were required to construct the opposite scenario: the animal was considered fixed, and the sensor (human observer) made lateral movements. On consecutive weekends in March and April 2021, we undertook five 24‐h cycles of boat‐based survey effort for a total of 120 observation hours (OH) with homogenous by‐hour coverage of 5 h per clock hour. Traveling along five 6‐km‐long transect lines, we recorded observations of *H. p. xanthos* at or within 3 m of the water surface. Each cycle was completed over two 12‐h sessions: one from ~05:45 h to ~17:45 h Saturday, followed by an overnight from ~17:45‐h Sunday evening to ~05:45‐h Monday morning. During each 24‐h cycle, we repeatedly traversed one transect, generally traveling at 6 kph (equating to about one hour of travel time per pass) with tight 180‐degree turns at each end. The observer remained at the prow scanning the water while the boat driver navigated using a handheld Garmin GPSMAP 64. As both crewmates were experienced at detecting snakes and accustomed to working extensive hours, we were able to sustain continuous observational effort through the sessions. During breaks for personal or logistical needs, which were infrequent (2–5 per 24‐h cycle) and short (average = 7.2 min), at least one crewmate maintained visual contact with the water. For the overnight sessions, LED lamps were attached to the bow, illuminating waters between 60‐degree port and starboard and to approximately 20‐m distance; headlamps and flashlights provided supplemental light. If a snake was seen near the end of a transect line, we took care to ensure a duplicate recording was not logged at the start of the next pass; although we could not confirm that individual snakes were never resighted in the middle of the transect line, the likelihood was extremely low because surface drift was never directly in line with the transect, so floating snakes were steadily carried away from our observation area. All snake sightings were marked in a handheld Garmin GPSMAP 64 and documented in a field log with date, time, and distance from boat. Every 30 minutes, we recorded environmental variables, including Beaufort Wind Force (BWF), prevailing weather (clear, cloudy, overcast, or raining), and SST was measured to 0.01°C using a traceable standard thermometer.

**FIGURE 1:**
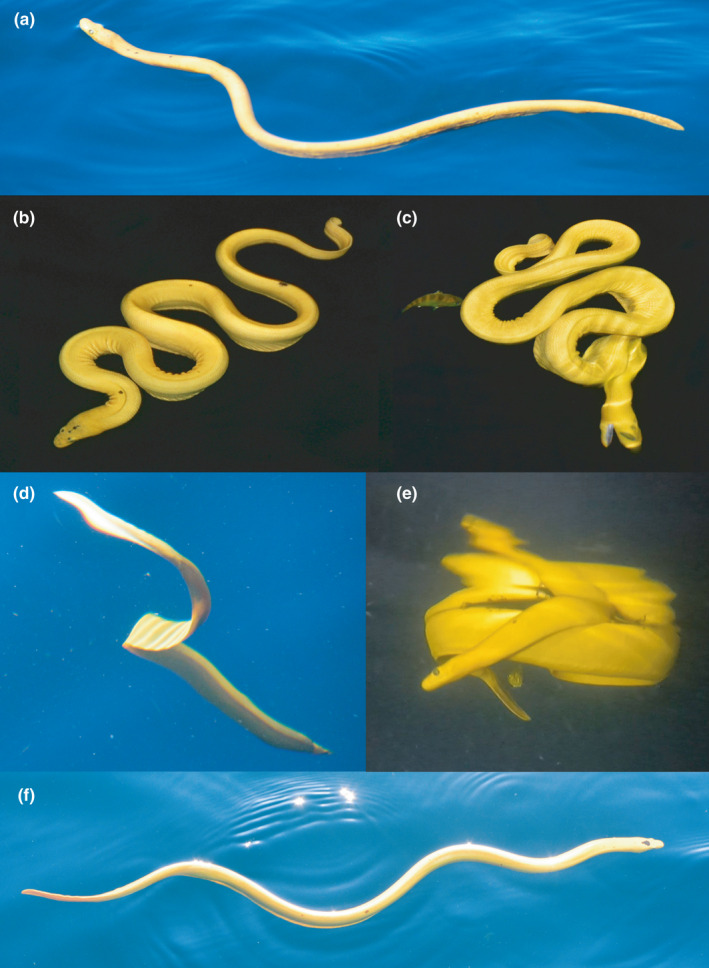
Ethogram of surface behaviors for *Hydrophis platurus xanthos*: (a) *RO*, resting open: floating loosely at the surface (the snake is also breathing here with nares above water); (b) *RS*, resting serpentine: floating in pronounced S‐shape; (c) *FS*, feeding sinusoidal: tight ambush posture, head pointing down; (d) *A*, avoidance: diving down (or arriving to the sea surface); (e) *K*, knotting: looping or coiling (seen here from below the surface with a light‐induced reflection); (f) *N*,*nadando*: swimming forward or backward. Procreation: breeding/birthing, not shown

Recorded surface behaviors for *H. p. xanthos* were coded using a pictorial ethogram (Figure 1). We recorded all distinct behaviors detected during each observation, but because observation periods were usually 15–90 s, in most cases, only a single behavior occurred. Feeding can occur when a snake is in its tight ambush posture, FS (feeding‐foraging sinusoidal) but also while swimming backward or from an open posture with the head held low and/or mouth open. For our purposes, any floating body position other than the ambush posture was considered “resting” with one of two categories of contraction registered: *RO* (resting open: floating in a looser or elongated posture) or *RS* (resting serpentine: contracted into a pronounced “S” shape). Other behaviors were *A* (arriving avoidance: seen <3‐m depth while arriving to the ocean surface or diving away), *K* (knotting: looping or coiling of the body), *N* (swimming [*nadando* in Spanish]: forward or backward movement across the sea surface via serpentine undulation), or *P* (procreation: breeding or birthing). Snakes found dead were classified as *D*.

### 2.4 Datasets and analyses

We analyzed two datasets: diel data (DD) represented hourly sightings collected during the 2021 diel survey, and nondiel data (NDD) comprised hourly effort and counts for 406 sightings of *H. p. xanthos* recorded outside the diel survey (Bessesen & González‐Suárez, 2021). The NDD came from other studies conducted between 2010 and 2021 and comprised both dry and rainy season encounters in the inner basin of Golfo Dulce (Bessesen, 2012, 2015, unpublished data). Using R version 4.0.3 (R Core Team, 2020), we employed circular statistics to analyze the datasets, with sightings binned into clock hours (24 bins) and day‐night boundaries set at 06:00 h and 18:00 h. Encounter rates were computed as sightings (counts) divided by observation hours (OH) within the snake's distribution area (effort) for each bin. We compared encounter rates for the DD and NDD to test if both supported the same patterns with Watson's two‐sample test of homogeneity using the watson.two.test function in the R library ‘circular’ (Agostinelli & Lund, 2017).

Focusing on the DD dataset, we tested the null hypothesis that observations were uniformly distributed across 24 h using the Rayleigh test of uniformity (rayleigh.test function in the R library ‘circular’) and the Hermans‐Rasson test (Hermans & Rasson, 1985), which has been found more reliable for data that follow a multimodal distribution (Landler et al., 2019). The Hermans‐Rasson test was completed using the code provided by Landler et al. (2019; Online Resource 3) with *p*‐values defined based on comparison with 1000 simulated uniform distributions. To identify times at which observations departed from the values expected given a uniform/random surfacing activity, we simulated 10,000 diel studies (using the rcircularuniform function in R) in which the total number of detected snakes and sampling effort (5 h per hour bin) were equal to those of the DD, but with observations randomly allocated to each hour. We then calculated simulated encounter rates and compared those values with observed rates in the DD and NDD. Observed values outside the 95% CI of the simulated rates were used to identify times at which snake sightings were significantly more or less frequent than expected if surfacing activity was similar over all hours of the day. Finally, we used the R package ‘activity’ (Rowcliffe, 2021) to estimate activity rates for both DD and NDD using a kernel bandwidth multiplier of 1.5 as suggested by Rowcliffe et al. (2014).

For each recorded ethogram behavior, we computed hourly frequencies and ran a Herman‐Rasson test in R to investigate nonuniformity in the diel pattern (as above, *p*‐values were defined comparing with 1000 simulated uniform distributions). We then investigated differences in behavior by assigning records into day and night periods (day was from 06:00 h and 18:00 h). Using these counts, we first compared behavior percentages for each period (counts of the behavior/total records for the periodic state). This helped us determine what snakes are more likely to do during the day or night. To test whether particular behaviors were significantly more or less likely to occur at night, we fitted a generalized linear mixed‐effects model (using the glmer function in R, with a binomial error structure) with day vs. night as the binary response and the observed surface behavior categories as a predictor. To control for temporal and spatial nonindependence of records obtained in the same transect and sampling period, we included transect line as an intercept random factor. We report model coefficients as odds ratios.

## 3 RESULTS

### 3.1 Field survey

**FIGURE 2:**
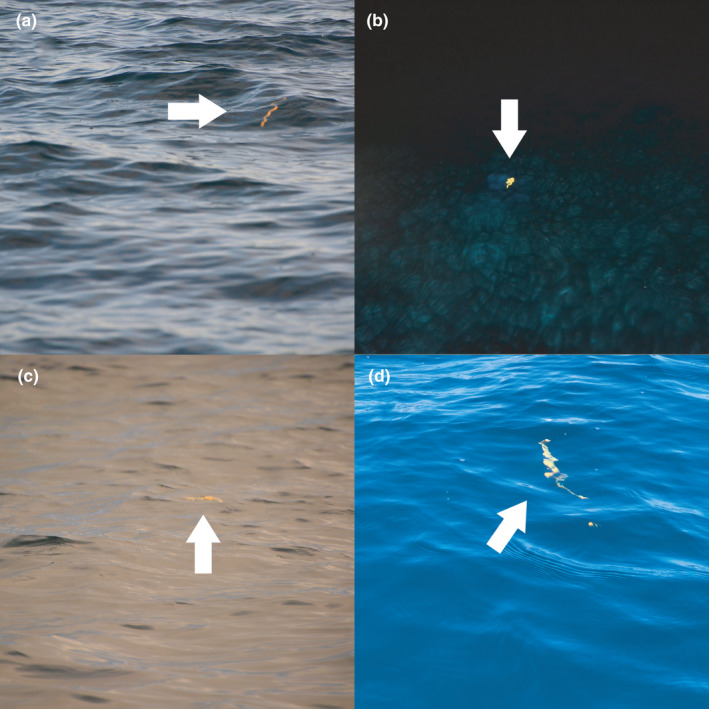
Detectability of *Hydrophis platurus xanthos* (pointed by white arrows) in various periodic states: (a) lifted by a late‐afternoon wave; (b) in spotlight at night (sinusoidal ambush posture); (c) in first sunrays of morning; and (d) diving midday (at <3 m depth)

During the diel survey, we logged a total of 358 sightings of *H. p. xanthos*. The skies above were most often cloudy but frequently became overcast with occasional rain. Mean SST across all hours of observation was measured at 29.2°C (Table 1; daytime = 29.4°C, nighttime = 28.9°C). The BWF ranged from 0 (glassy) to 5 (moderate waves, some spray), and snakes were recorded in all except BWF0. Because data were collected over multiple weekends and times, environmental conditions and solar exposure varied (Figure 2), but we found *H. p. xanthos* to be consistently detectable at or near the sea surface. Approximately 200 sightings of the NDD had been previously modeled in the multicovariate distance sampling engine of Distance 7.3 (Thomas et al., 2010) to test the effect of time of day and/or BWF on detection by distance, and no effect was found (*p *= .20–.48). Sometimes two or more detections of *H. p. xanthos* occurred close to each other (<50 m) and in a matter of minutes, although individuals did not interact with one another and were rarely closer than 15 m. With the boat moving slowly and at a consistent speed, the animals showed no reaction to our presence. All sightings occurred within 20 m of the boat, even during the day when overall visibility could be >25 km, mitigating concerns of perception bias between day and night.

**TABLE 1:** Survey data information for each transect line, including the survey date, observation hours, total sighting counts for day and night, prevailing weather, minutes of rainfall, and average sea surface temperature

Transect	Dates	Hours	Total day	Total night	Weather	Rain	SST
TL3	Mar 27–29	24	8	33	C, X	0	30.48
TL2	Apr 3–5	24	19	74	C, O, R	120	28.62
TL1	Apr 10–12	24	16	114	O, C, R	45	28.59
TL4	Apr 17–19	24	6	57	O, C, R	165	28.43
TL5	Apr 24–26	24	9	22	C, O, R	195	29.71
All		120	58	300		525	29.16

Note

Day: 06:00–18:00 h; night: 18:00–06:00 h. X, clear/no clouds; C, clouds; O, overcast; R, rain; listed in order of recorded frequency. Bottom line (All) calculates column tallies, except for SST shown as mean.

### 3.2 Diel surfacing pattern

**FIGURE 3:**
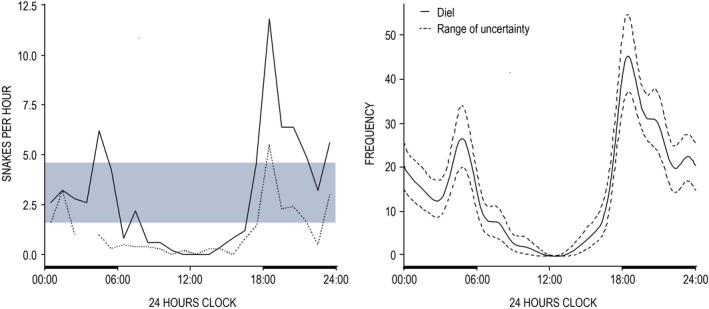
Diel data on 24‐h clock (bold *x*‐axis line: night 18:00 h and 06:00 h). LEFT: Observed by‐hour encounter rates of diel data (solid line; consistent effort with five 24‐h cycles) and nondiel data (dashed line; opportunistic records with variable effort and no surveys from 03:00–04:00 h). Gray ribbon shows 95% confidence intervals of simulated uniform encounter rates. RIGHT: Estimated by‐hour surfacing probability rates (mean and 95% CI) of *Hydrophis platurus xanthos*

During the diel survey, only 16% (*n* = 58) of *H. p. xanthos* sightings occurred during the day (06:00–18:00 h) with a significant departure from uniform surfacing activity (Rayleigh test statistic = 0.46, *p *< .001; Hermans‐Rasson test statistic = −649.47, *p* < .001). Compared with simulated uniform observations, snakes were significantly less often found at or within 3 m of the sea surface between 08:00 h and 16:00 h but were more frequent than expected between 04:00 h and 05:00 h and from 18:00 h to 21:00 h (Figure 3). This pattern corresponds to crepuscular‐nocturnal activity, with the highest level of surfacing detected post sunset. Noticeably during the diel survey, no sightings were logged between the daytime hours of 11:00 and 14:00 (15 h of observation with no sightings). Hourly survey effort was estimated for the NDD and varied: no surveys were conducted from 03:00 h to 04:00 h, and for other times, effort ranged from 1 to 23.5 OH (average = 14). Despite the variable sampling effort, encounter rates from the 406 sightings of the NDD had a similar pattern to those of the DD data (Watson's two‐sample test of homogeneity statistic = 0.15, *p *> .10) supporting crepuscular‐nocturnality, although rates were overall lower (Figure 3).

We estimated the mean activity rate (proportion of time at surface) from the DD for *H. p. xanthos* to be 0.330 (SE = 0.0337), and hourly probability rates varied with the observed crepuscular‐nocturnal pattern (Figure 3). The NDD rendered a lower mean activity rate of 0.177 (SE = 0.0140), reflecting fewer sightings overall during the studies from which the NDD were compiled, as well as gaps in survey effort with scant nighttime coverage, especially between 23:00 h and 06:00 h. Although diurnal sightings were sporadic, more were present in the NDD owing to much greater daytime sampling effort (up to 23.5 OH per clock hour). We note that these activity rates could be overestimated because the model assumes all snakes are at the surface during the peak hour (Rowcliffe et al., 2014; Rowcliffe, 2021), which might not be true. Foraging would be unlikely to draw all numbers to the surface at once since snakes do not normally eat every day (Greene, 1997; Lillywhite, 2014). Yet, it is still possible that most or all *H. p. xanthos* surface when the sun goes down, if for the sole purpose of resting after a day of dive cycles, reducing energy expenditure through positive buoyancy (Graham et al., 1975). Sighting surveys across both seasons suggested higher encounter rates around dusk and dawn, although we did find differences in surface activity patterns between the dry season (2010 and 2020; *n* = 87 records) and rainy season (2011; *n* = 37; Watson's two‐sample test of homogeneity statistic = 0.31, *p *< .01). This may reflect true temporal variation or the relatively small size of the rainy season sample and discrepancies in hourly survey effort between years (for example, when considering the period between 13:00–24:00 h, in the dry season, we logged 2–19 OH per clock hour but ≤1 OH per clock hour in the rainy season).

### 3.3 Surface behaviors

For the ethogram‐coded behaviors (Figure 1), we recorded a total of 470 data points in order of frequency: *RO* (*n* = 213), *RS* (*n* = 126), *A* (*n* = 47), *FS* (*n* = 38), *N* (*n* = 38), and *K* (*n* = 8); no procreative behavior or dead snakes were observed. For most snakes, we observed a single behavior, but for about a quarter of individuals (*n* = 92), we recorded 2–4 different behaviors within the brief period of observation allowed as we passed. Snakes were equally likely to exhibit multiple behaviors by day or night (26% of sightings in both periods). All six behaviors were significantly nonuniform (Herman‐Rasson tests: A statistic = −78.57, *p *= .024; FS statistic = −73.01, *p *= .011; *K* statistic = −25.94, *p *= .013; *N* statistic = −86.78, *p *= .002; RO statistic = −377.43, *p* < .001; *A* statistic = −295.08, *p* < .001).

**FIGURE 4:**
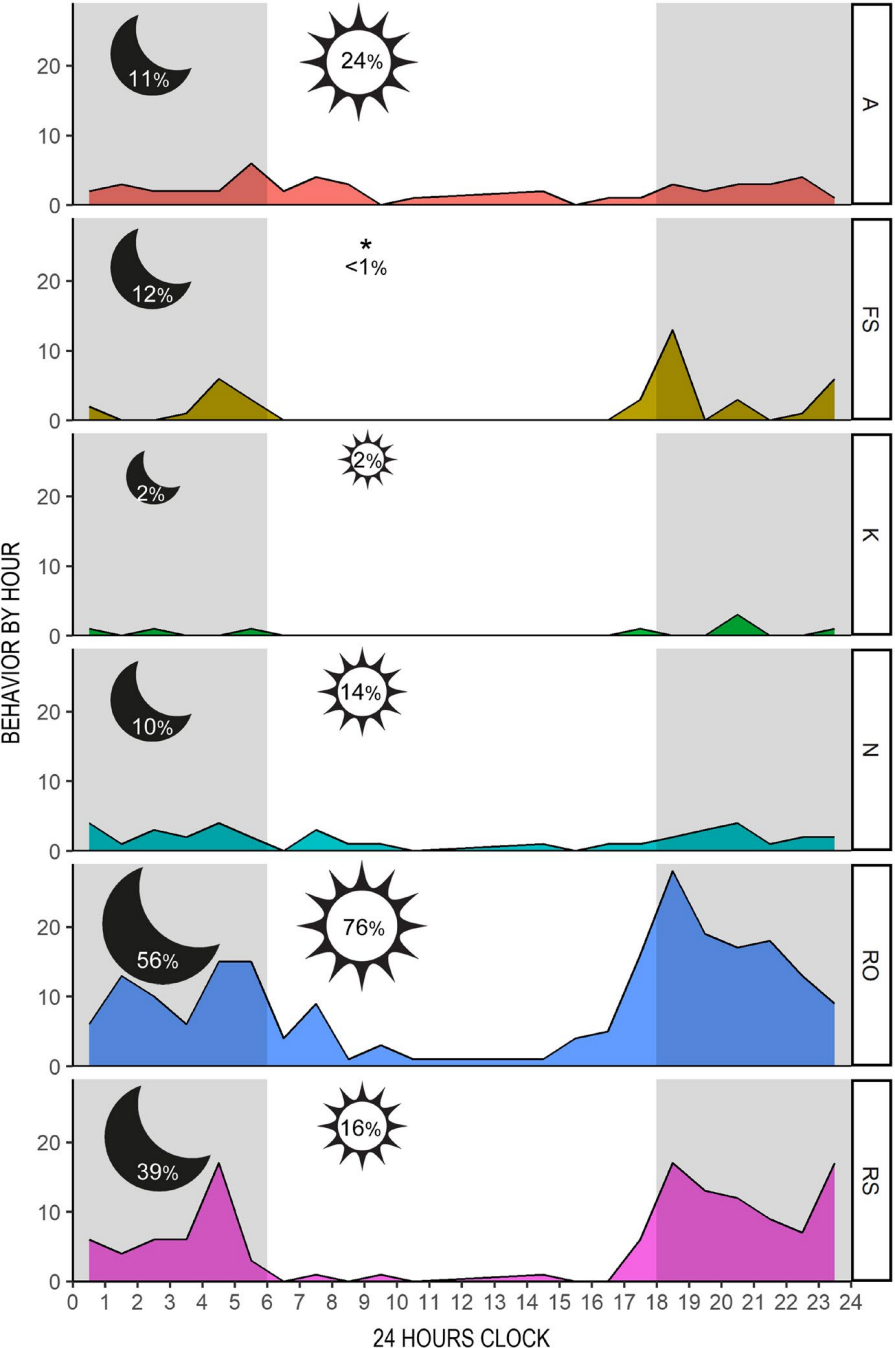
Area graphs for by‐hour frequencies of surface behaviors of *Hydrophis platurus xanthos*:*A* (diving or surfacing), *FS* (feeding sinusoidal), *K* (knotting), *N* (swimming), *RO* (resting open), and *RS* (resting serpentine); percentages of total recorded behaviors by period are shown inside moons (night) and suns (day), scaled for quick reference (larger icons for higher percentages)

Sea snake behavior varied between night and day (Table 2; Figure 4). For example, nearly a quarter of sea snakes found during the day were diving and surfacing (*A*) compared with only 11% of those found at night. Floating in an open or loose body posture (*RO*) also appeared a more common daytime behavior. Conversely, resting in a serpentine shape (*RS*) and feeding sinusoidal (*FS*) were far more common at night, when snakes fluidly shifted between *RO* and *RS*, and sometimes in and out of *FS*. As previously reported by Bessesen and Galbreath (2017), we only saw *H. p. xanthos* in its ambush posture (*FS*) between sunset and sunrise (3 sightings of *FS* occurred post sunset but within 9 min of 18:00 h). Snakes were found swimming (*N*) and knotting (*K*) in similar percentages during day and night (Figure 4).

**TABLE 2:** Odds ratios for nighttime observation of surface behaviors of *Hydrophis platurus xanthos* based on a generalized linear mixed‐effects model (glmer function in R, family binomial, day and night as binary responses, observed surface behaviors as predictors, transect line as random factor modifying the intercept)

	OR	CI
Diving surfacing (*A*)	2.43	1.212–4.888
Feeding sinusoidal (*FS*)	10.78	3.188–36.458
Knotting (*K*)	6.26	0.746–52.482
Swimming (*N*)	3.86	1.664–8.949
Resting open (*RO*)	3.63	2.314–5.684
Resting serpentine (*RS*)	12.62	5.994–26.588

Odds ratios provided an intuitive way to interpret our model results (Table 2). For all 470 records, behavior was 4.7 times (SE = 1.23) more likely to occur at night than during the day. This value provides a reference for comparison with OR estimates for different behaviors and shows *FS* and *RS* to be primarily nocturnal behaviors. *FS* was 10.78 times more likely to occur at night, more than twice the average likelihood, and *RS* was nearly three times the average (OR = 12.62). Because we considered all observed behaviors and sometimes more than one behavior was observed in the same snake, our model results could have been influenced by pseudoreplication, and because we recorded a single behavior for most snakes, using snake ID as a random factor (to control for nonindependence) resulted in convergence issues. Confidence was gained, however, when a model that considered only the first recorded behavior for each snake showed the same qualitative results.

## 4 DISCUSSION

Confined to the deep inner‐basin waters of Golfo Dulce, *H. p. xanthos* appears to have developed a defined multimodal circadian rhythm. Its surfacing pattern is one of the crepuscular‐nocturnality, described by more observable surfacing at night with conspicuous peaks in the twilight hours. Nocturnality was patterned by some surface behaviors too. Notably, the taxon's unique ambush posture and seemingly associated position of resting serpentine are almost exclusively seen at night, when individuals fluidly transition between resting, foraging, swimming, and knotting. While daytime sightings of *H. p. xanthos* are rarer, they do occur. In fact, the reported diel surfacing pattern is more likely to reflect duration of time at the surface than number of surfacing events. Given the need to breathe, sea snakes undoubtedly surface repeatedly throughout the day (diving and surfacing actually represented a higher percentage of behavior records during the day than at night), but because they can take 1–2 breaths and resubmerge in a matter of seconds (Myers, 1945; Rubinoff et al., 1986, personal observation), the chance of detection is greatly reduced. Every observed dive required active propulsion, but surfacing did not always occur through lateral undulation. Some snakes rose up through the water column while seemingly motionless and horizontally positioned, the maneuver ostensibly managed through buoyancy. The most common surface behavior in both principal periodic states was resting open; it accounted for more than half of the nighttime and three‐quarters of the daytime records. Two behaviors appeared equally common in daylight or darkness: swimming and knotting, granting the sample size of the latter was too small for confidence.

Because all *H. p. xanthos* reside in one small and relatively sheltered marine habitat, we were able to identify patterns for the entire population, although it is worth noting that we did occasionally find snakes floating at the surface during atypical hours. That two or more snakes sometimes surfaced in the same general area within a narrow time frame, even during the day, suggest that common factors drive surfacing, and pockets of conducive underwater conditions may help explain proximal groupings. Even in peak hours, multiple factors likely influence behavior and length of time at the surface, including environmental conditions, physiological needs, and prey availability (Daltry et al., 1998; Helfman, 1986), which could be explored in future studies. Although we focused on the diel scale, other biological clocks besides circadian (tidal, lunar, and seasonal) may also affect behavioral patterns (Udyawer et al., 2017, 2020). Seasonal comparisons in the NDD suggested some temporal difference, but the reliability of that finding is uncertain due to the small sample size and limited survey effort. It is possible that cloud cover during the rainy season and a slightly shorter photoperiod allows *H. p. xanthos* to extend its surfacing hours into early post sunrise in the morning and/or late afternoon. Still, the basic pattern appeared to hold year‐round and over a decade of observations, and we would not expect it to change significantly given that tropical regions are relatively stable, and the snakes are obligated to regularly breathe, eat, and drink at the water surface in the periodic state most suitable for their survival.

Temperature plays a prominent role in snake biology (Greene, 1997; Weatherhead & Madsen, 2009), and while rainfall appears to have little effect on the activity of tropical snakes (Brown & Shine, 2002), overcast skies and rain bring cooler temperatures. Our survey ended in April, the last month of the dry season in Costa Rica, and precipitation became progressively heavier and more frequent through the study period. Although SST does not fully describe the thermal environment, we measured an average of 29.2°C, with the daytime average only 0.3°C higher than at night. Importantly, at the height of the dry season, SST in Golfo Dulce can surpass 32°C (Rincón‐Alejos & Ballestero‐Sakson, 2015, Bessesen 2015), and while the critical thermal maximum for *H. p. xanthos* is unknown, its parent taxon, *H. p. platurus*, has a reported maximum of 33–36°C (Dunson & Ehlert, 1971; Graham et al., 1971). A black dorsum collects heat when exposed to sunlight (Graham, 1974), and diurnal foraging naturally increases that exposure. It was almost certainly temperature that drove *H. p. xanthos* to evolve its nearly all‐yellow coloring. It has already been suggested that cooling in xanthic sea snakes is promoted by lighter skin color (Bessesen, 2012; Solórzano, 2011), and a smaller body size (Bessesen & Galbreath, 2017) increases the surface‐area‐to‐mass ratio for more rapid thermal exchange (Ashton & Feldman, 2003). We hypothesize that *H. p. xanthos* could also have functionally transitioned to warmer waters by reducing its surfacing time during the day, shifting from diurnality to nocturnality to avoid overheating. Despite these morphologic and behavioral adaptations, if *H. p. xanthos* is surviving near the top of its thermal limit, the trend of warming SST in Golfo Dulce, as recently reported by Murayama et al. (2018), could threaten long‐term survival of the population.

Temperature, however, might not be the only, or even the main, driver of nighttime surfacing by *H. p. xanthos*. Melanin protects a squamate's integument and internal organs against the damaging effects of ultraviolet radiation (Greene, 1997; Lillywhite, 2014; Porter & Norris, 1969). Dark skin over venom glands may specifically protect venom from degradation (Pough et al., 1978). Thus, lacking the protective melanin of *H. p. platurus*,*H. p. xanthos* may be photosensitive, and if so, nocturnality could mitigate tissue damage from solar radiation and preserve venom potency for successful feeding. We further hypothesize that the observed postsunset peak in surfacing could be caused by energetic needs. Tiny fish are frequently seen in association with *H. p. xanthos* at sightings around the clock, and snakes do sometimes feed when the sun is above the horizon (from an RO posture; personal observation), but if solar radiation and/or other factors limit the duration of daytime surfacing events, snakes could become increasingly hungry while waiting until the sun sets before floating for prolonged foraging periods. While fish also evince diel patterns (Helfman, 1986), *H. p. xanthos*' prey may be regularly present: one of more fish are commonly observed alongside, ascending, and descending in the water column (unpublished data), though the snakes never feed at depth. The snake's sinusoidal ambush posture likely developed to accommodate for ocean turbulence, which commonly increases in Golfo Dulce in the late afternoon and evening (personal observation).

A potentially important benefit of nocturnal surfacing could be predator avoidance. The parent species, *H. p. platurus*, is advantaged by both countershading (Graham et al., 1971) and aposematism, with no known natural predators (Kropach, 1975). Whether transitioning to a yellow dorsum in *H. p. xanthos* has bearing on rates of predation is unknown; however, these snakes do appear timider than their black‐backed conspecifics (Bessesen, 2012). Xanthic snakes are known to be harassed by dolphins (Bessesen et al., 2021). They are also occasionally found with scars that indicate traumatic injury, including one with a missing eye (unpublished data). Since several known avian predators are present in Golfo Dulce (pelicans, ospreys, black hawks, and magnificent frigates), nocturnal surfacing could mitigate the risk of incidental attack. Avoidance of boat traffic in the embayment might be another added benefit of nighttime activity, though regular daytime surfacing for ventilation could still put snakes at risk. Xanthic sea snakes rarely if ever dive when a boat drives near or even over them, apparently ignorant to the mortal danger of propeller strikes (personal observation), but we have noticed that during the day, snakes are more likely to react to loud, sudden noises nearby such as a boat motor starting or thumps inside the hull and will sometimes swim forward with the head lifted well above the water to presumably make observations before quickly diving away (the retinal structure of *H. p. platurus* suggests visual acuity below and above the water; Hibbard & Lavergne, 1972).

Crepuscularity is rare among sea snakes (Simões et al., 2020). As previously stated, *H. p. platurus* is considered diurnal (Brischoux & Lillywhite, 2011; Kropach, 1975; Rubinoff et al., 1986; Simões et al., 2020), though Lillywhite et al. (2015) noticed a more nuanced pattern that emphasized morning surfacing (between 07:00–11:00 h; also see Tu, 1976). While Udyawer et al. (2015) found free‐ranging congener species, *H. curtus* and *H. elegans*, to be more active at the water surface at night, Heatwole and Seymour (1975) studied *H. elegans*,*H. peronii*, and *H. belcheri* in the laboratory and found all three to be less active at night. Simões et al. (2020) inferred diel activity patterns for several sea snakes using genetic variation in spectral sites, trawl bycatch data and previous literature. They identified *H. elegans* as cathemeral (which might explain the contradictive findings between field and laboratory) and confirmed *H. curtus* and *H. peronii* as diurnal; *H. belcheri* was not categorized. Of the 17 species from the genus *Hydrophis* for which a diel pattern was included, similar numbers were reported as cathemeral (*n* = 5), nocturnal (*n* = 5), and diurnal (*n* = 7), yet even with an explicit fourth category, none were reported as crepuscular. That *H. p. xanthos* breaks from the patterns of its closest relatives poses an interesting evolutionary question and calls attention to the limits of inferring the behavior of understudied taxa.

Garnering behavioral and activity data is not easy (Bridges & Noss, 2011), especially on a population level and under natural conditions. However, such studies are needed. Transmitters are a common tool for securing activity and spatial data. These devices are typically attached to or implanted in the bodies of anesthetized snakes through surgical methods (as in Rubinoff et al., 1986; Shetty & Shine, 2002; Udyawer et al., 2015) or swallowed (as in Burns & Heatwole, 1998; Weatherhead & Blouin‐Demers, 2004) but can have negative impacts on immunity, fecundity, and lifespan (see Riley et al., 2017 for a review). Tag studies are also expensive and sample sizes tend to be small (Lashley et al., 2018), plus resulting changes in behavior can bias results (Fitch & Shirer, 1971). Camera trapping is slowly gaining ground as a method for detecting terrestrial snakes, but diel patterns have yet to be published, and the potential for use in the marine environment is yet undetermined (Neuharth et al., 2020; Welbourne et al., 2017). Unmanned underwater object tracking devices show real potential for collecting movements and behavioral data but currently require a transponder attached to the animal (Dodge et al., 2018; such equipment is easily suction‐cupped to a sea turtle shell but less applicable for a sea snake). New marine research tools continue to emerge, from passive acoustic monitoring to side‐scan sonar to remote sensor satellites, and with collaborative pioneering between ecologists and engineers, improved techniques for recording the behavior and activity patterns of sea snakes are likely to be developed. In the meantime, the value of noninvasive if arduously collected observational data should not be underestimated and can be particularly useful in taxa that, like *H. p. xanthos*, are observable and inhabit narrow, navigable ranges. Real‐time visual diel surveys are especially advantageous when dealing with small, endemic, or otherwise vulnerable populations, which could suffer from invasive or potentially detrimental methods.

There is an intensifying need to facilitate the research and monitoring of sea snakes around the world. The International Union for Conservation of Nature identifies approximately a third of species as data deficient (Elfes et al., 2013; IUCN, 2021), and of those studied, many are reported in inexplicable decline (Goiran & Shine, 2013; Lukoschek et al., 2013; Udyawer et al., 2018). Among the fundamental ecological data required to inform protection strategies, established diel patterns not only enable improved survey designs but may also serve as indicators since altered or declining activity can reveal negative trends (Barrueto et al., 2014). Our work therefore supports the conservation of *H. p. xanthos*. It also contributes to the overall understanding of this endemic subspecies, which, given genetic isolation and the accumulative evidence of morphologic and behavioral distinctiveness, may warrant recognition as a new species.

## CONFLICT OF INTEREST

The authors declare no conflict of interest.

## AUTHOR CONTRIBUTIONS

**Brooke Bessesen:** Conceptualization (lead); Data curation (lead); Formal analysis (lead); Investigation (lead); Methodology (lead); Project administration (lead); Writing – original draft (lead); Writing – review & editing (equal). **Manuela Gonzalez‐Suarez:** Conceptualization (supporting); Formal analysis (supporting); Investigation (supporting); Methodology (supporting); Supervision (lead); Validation (lead); Writing – review & editing (equal).

## Data Availability

Data analyzed for this article are available through the public repository Figshare, doi: https://doi.org/10.6084/m9.figshare.17052407.v1.
